# Improving methodology to determine vitamin D_3_ in food supplements

**DOI:** 10.1080/07853890.2021.1897427

**Published:** 2021-09-28

**Authors:** Carla Ferreira, Alexandra Figueiredo, Guilhermina Moutinho, Maria D. Auxtero, Isabel Margarida Costa, Alexandre Quintas

**Affiliations:** a Laboratório de Ciências Forenses e Psicológicas Egas Moniz, Centro de Investigação Interdisciplinar Egas Moniz (CiiEM), Egas Moniz Cooperativa de Ensino Superior, Caparica, Portugal; b Molecular Pathology and Forensic Biochemistry laboratory, Centro de Investigação Interdisciplinar Egas Moniz (CiiEM), Egas Moniz Cooperativa de Ensino Superior, Caparica, Portugal; c PharmSci Lab, Centro de Investigação Interdisciplinar Egas Moniz (CiiEM), Egas Moniz Cooperativa de Ensino Superior, Caparica, Portugal

## Abstract

**Introduction:**

Nowadays, there is a trend to enrich food and food supplements (FS) with Vitamin D_3_. Economic operators who places FS on the market does not have to perform running safety trials, but only to comply with the food safety regulations applicable in the European Union [1]. This scenario may present public health and legal issues. To overcome these challenges, it is mandatory to know accurately the amount of vitamin D_3_ in FS and if it corresponds to the label value. HPLC methods are a preferential approach to determine accurately the content of vitamin D_3_ in pharmaceuticals and supplements. Actually, HPLC/MS has become the technique of choice for vitamin D_3_ determination in complex matrixes. Notwithstanding, this analysis must be preceded by time-consuming sample preparation. In fact, the success to measure accurately vitamin D_3_ depends heavily in a reliable sample preparation. Moreover, it becomes even more critic when dealing with different formulations of vitamin D_3_, such has gel pills, solid pills, liquid or cutaneous applications. There are literature validating methodology to determinate vitamin D_3_ in various matrixes according to the International Conference on Harmonisation (ICH) guidelines by HPLC-UV [2]. However, this published study does not use an internal standard (IS). The internal standard is useful to improve the precision of quantitative analysis, removing the error of losing sample during sample preparation. Our aim is to test o-cresol as an internal standard to future validation the methodology.

**Materials and methods:**

Vit D_3_ present in FS was analysed by HPLC/DAD. Sample was spiked with internal standard (*o*-cresol) and liquid extraction coupled to an ultrasound bath was used has an extraction procedure. After a centrifuged step the supernatant was filtered. The separation was performed using a C18 column with a solvent gradient consisted in (A) 0.1% formic acid and (B) acetonitrile. The acquisition was performed on DAD-detector at 265 nm.

**Results:**

Figure 1 shows a chromatogram of a FS containing Vit D_3_. Two major peaks are present, corresponding to *o*-cresol (RT 5.45 min) and Vit D_3_ (RT 11.20 min). **Discussion and conclusions:** The results show the capacity of the chromatographic method to separate and resolve the two chromatographic peaks. These preliminary results show that o-cresol can be used as an internal standard to determine Vit D3 in FS, normalising the loss of Vit D3 during preparation step. Since in Europe and USA, the association of vitD toxicity with the use of FS has been described, this methodology will be useful to confirm the VitD composition stated on the label.

**Figure 1.**
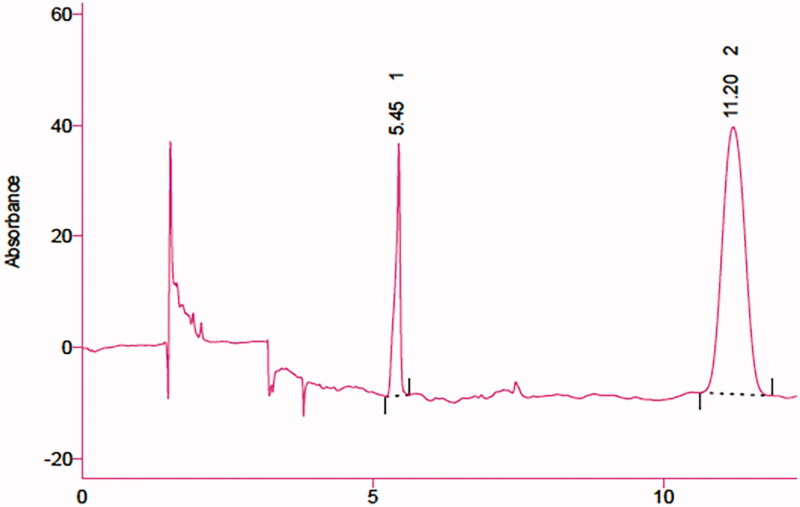
HPLC/DAD chromatogram of FD with IS (RT = 5.45 min) and Vit D_3_ (tr = 11.20 min).
